# Plantar Hyperidrosis

**Published:** 1914-10

**Authors:** 


**Plantar Hyperidrosis.** Sabatié ("Paris Médical") recommends the following treatment for perspiration of the feet:—

1. Bathe the feet daily, using hot or cold water according to circumstances, with the addition of a tablespoonful of formaldehyde solution.
2. Wash the feet twice daily with the following lotion:—Tinct. benzoin 10.0 gm.; Liq. formaldehyd., 40 per cent. 15.0 gm.; Aquam, ad 1.0 litre.
3. Change the socks daily, and wear the same boots only once in five or six days.
4. Place each morning in the socks the following powder:—Acid. salicyl., 1.0 gm.; Pulv. iridis, 10.0 gm.; Pulv. cret. gall., 40.0 gm. For treatment of bromidrosis, see Prescriber, 1912, p. 284.

